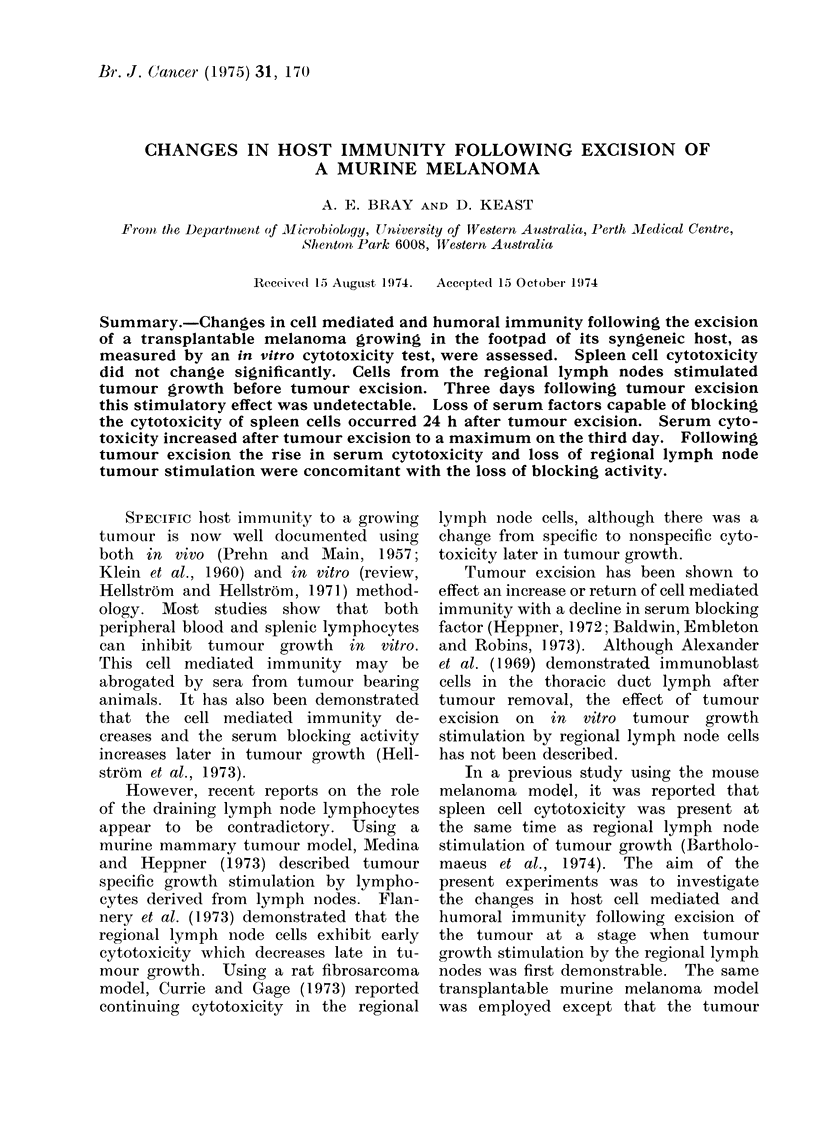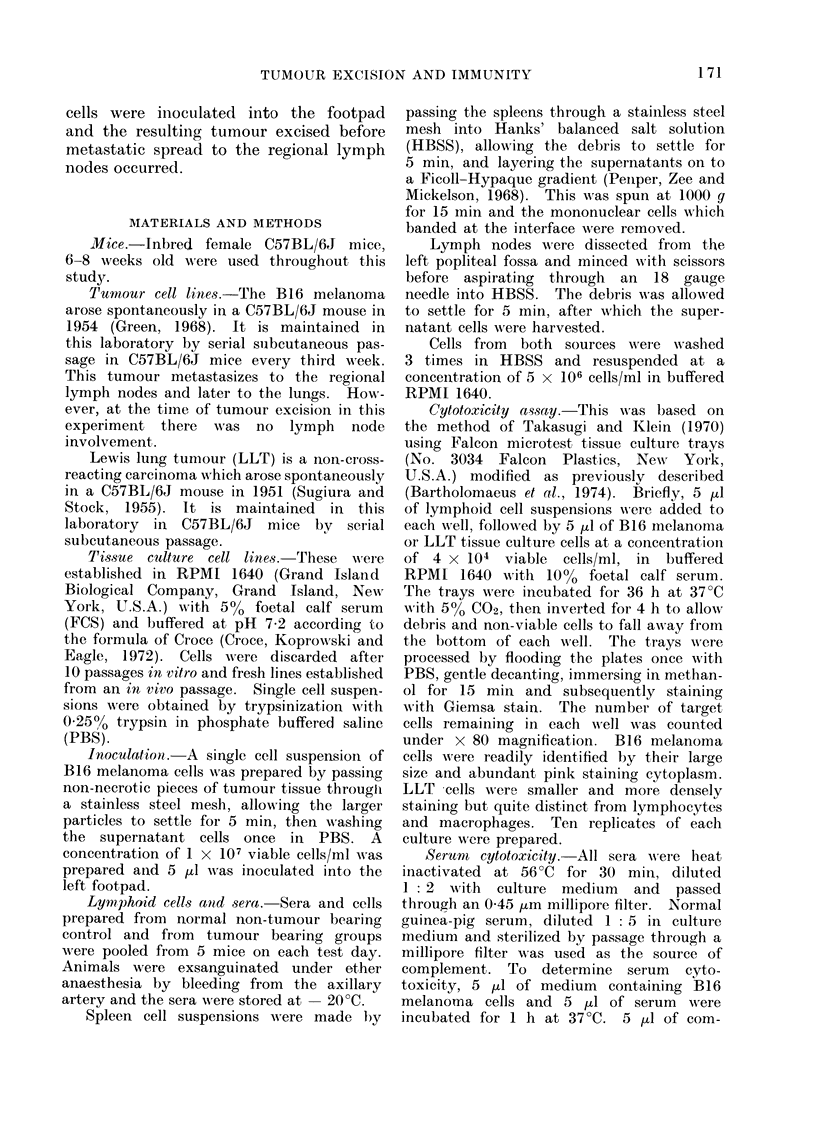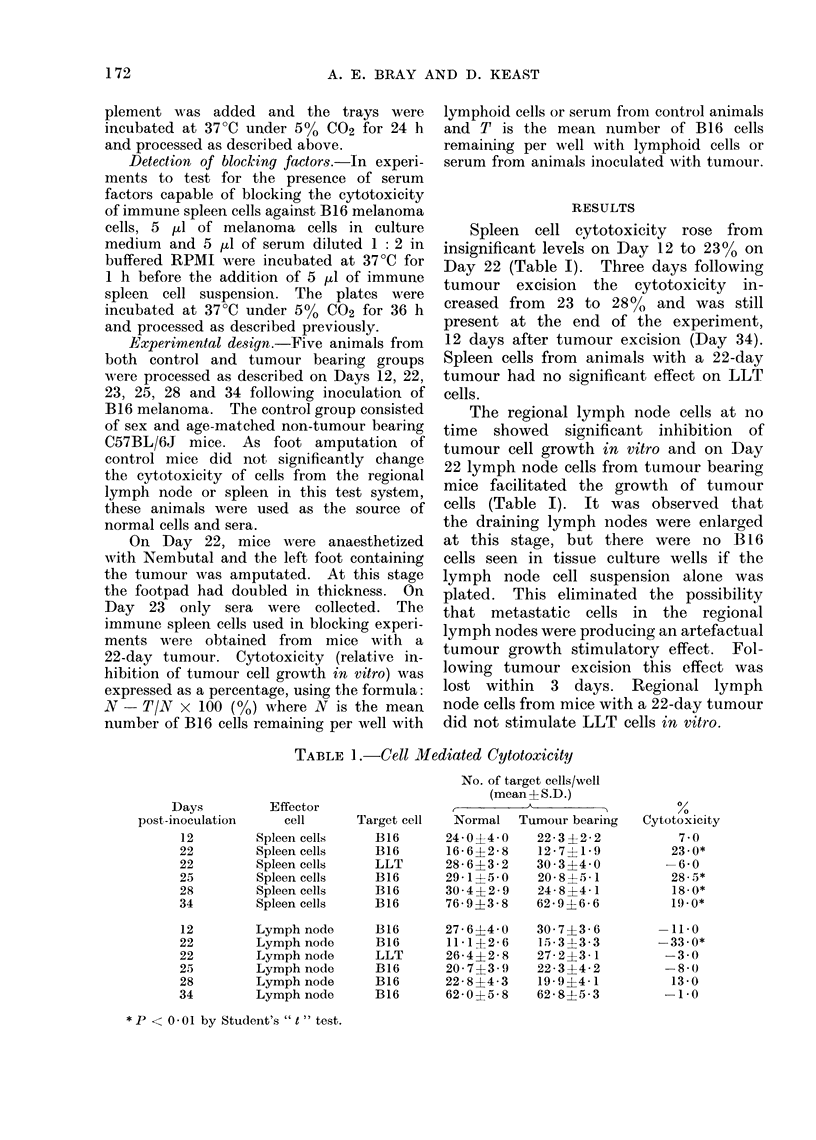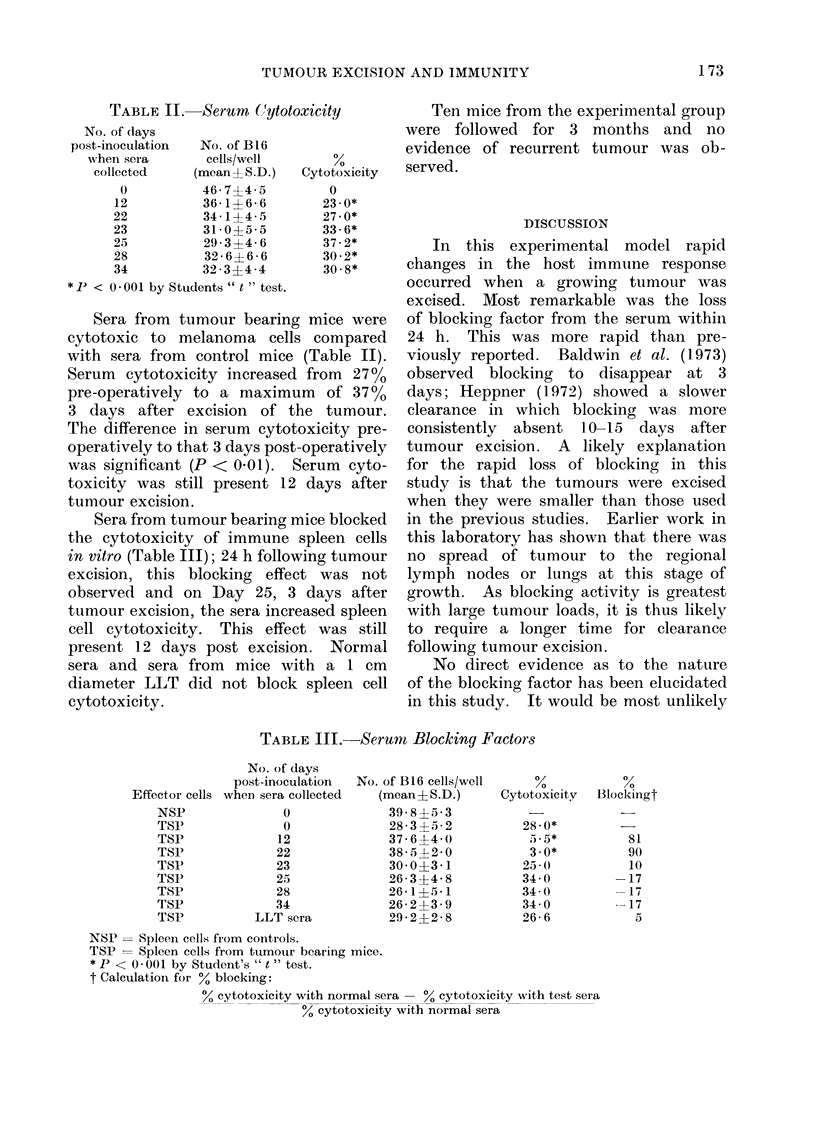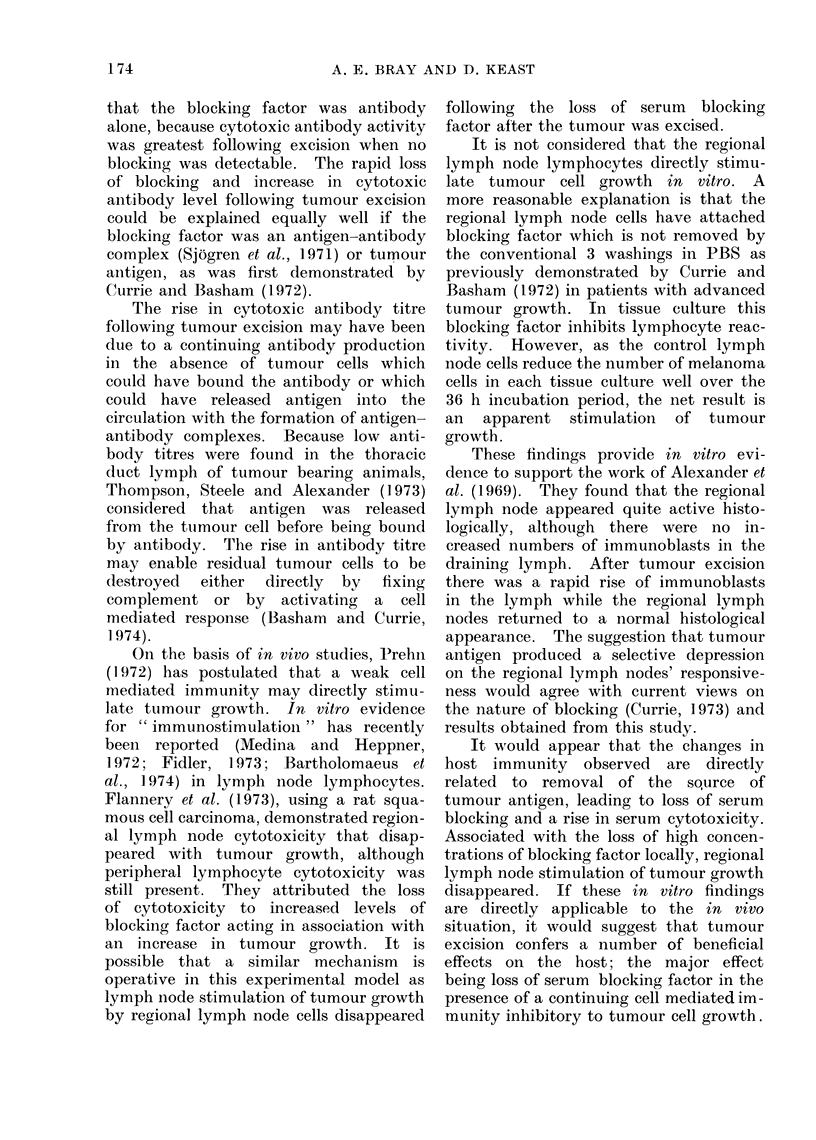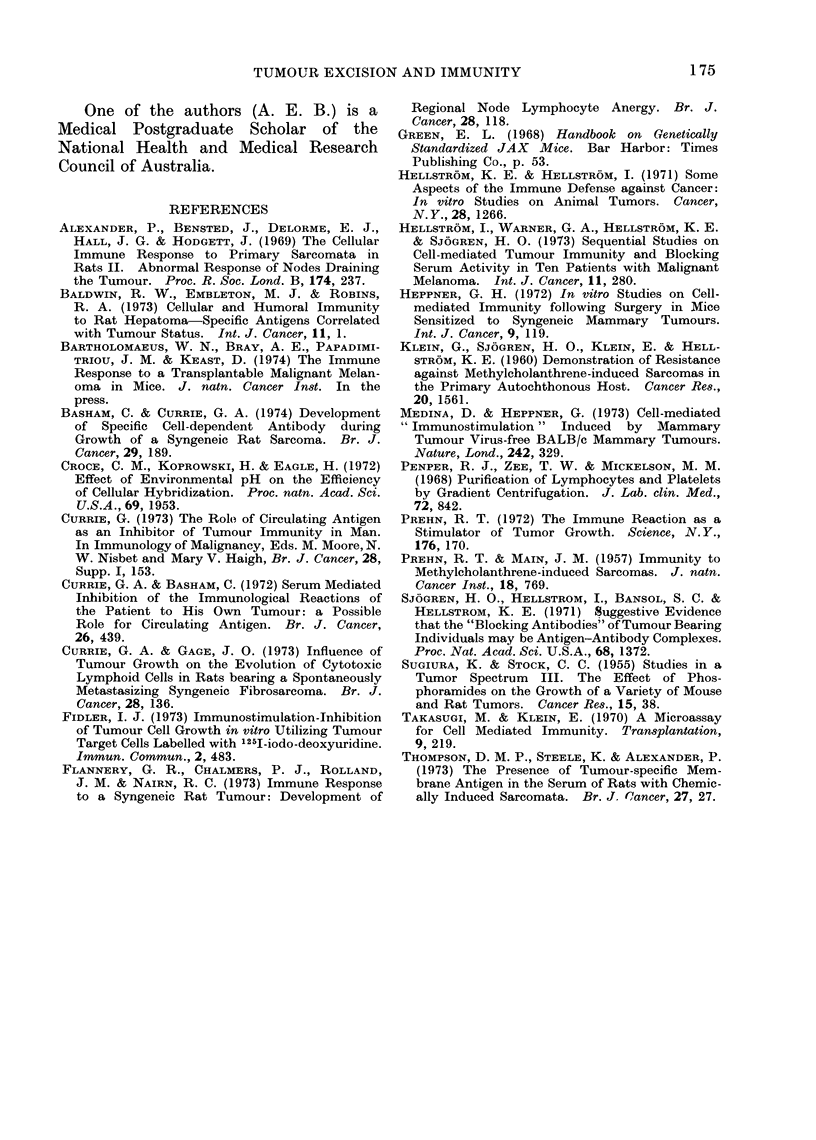# Changes in host immunity following excision of a murine melanoma.

**DOI:** 10.1038/bjc.1975.22

**Published:** 1975-02

**Authors:** A. E. Bray, D. Keast

## Abstract

Changes in cell mediated and humoral immunity following the excision of a transplantable melanoma growing in the footpad of its syngeneic host, as measured by an in vitro cytotoxicity test, were assessed. Spleen cell cytotoxicity did not change significantly. Cells from the regional lymph nodes stimulated tumour growth before tumour excision. Three days following tumour excision this stimulatory effect was undetectable. Loss of serum factors capable of blocking the cytotoxicity of spleen cells occurred 24 h after tumour excision. Serum cytotoxicity increased after tumour excision to a maximum of the third day. Following tumour excision the rise in serum cytotoxicity and loss of regional lymph node tumour stimulation were concomitant with the loss of blocking activity.


*Br. J. Cancer* (1975) **31**, 170

### CHANGES IN HOST IMMUNITY FOLLOWING EXCISION OF A MURINE MELANOMA

A. E. BRAY AND D. KEAST

*From the Department of Microbiology, University of Western Australia, Perth Medical Centre, Shenton Park 6008, Western Australia*

Received 15 August 1974. Accepted 15 October 1974

**Summary.—Changes in cell mediated and humoral immunity following the excision of a transplantable melanoma growing in the footpad of its syngeneic host, as measured by an *in vitro* cytotoxicity test, were assessed. Spleen cell cytotoxicity did not change significantly. Cells from the regional lymph nodes stimulated tumour growth before tumour excision. Three days following tumour excision this stimulatory effect was undetectable. Loss of serum factors capable of blocking the cytotoxicity of spleen cells occurred 24 h after tumour excision. Serum cytotoxicity increased after tumour excision to a maximum on the third day. Following tumour excision the rise in serum cytotoxicity and loss of regional lymph node tumour stimulation were concomitant with the loss of blocking activity.**

SPECIFIC host immunity to a growing tumour is now well documented using both *in vivo* (Prehn and Main, 1957; Klein *et al.*, 1960) and *in vitro* (review, Hellström and Hellström, 1971) methodology. Most studies show that both peripheral blood and splenic lymphocytes can inhibit tumour growth *in vitro*. This cell mediated immunity may be abrogated by sera from tumour bearing animals. It has also been demonstrated that the cell mediated immunity decreases and the serum blocking activity increases later in tumour growth (Hellström *et al.*, 1973).

However, recent reports on the role of the draining lymph node lymphocytes appear to be contradictory. Using a murine mammary tumour model, Medina and Heppner (1973) described tumour specific growth stimulation by lymphocytes derived from lymph nodes. Flannery *et al.* (1973) demonstrated that the regional lymph node cells exhibit early cytotoxicity which decreases late in tumour growth. Using a rat fibrosarcoma model, Currie and Gage (1973) reported continuing cytotoxicity in the regional lymph node cells, although there was a change from specific to nonspecific cytotoxicity later in tumour growth.

Tumour excision has been shown to effect an increase or return of cell mediated immunity with a decline in serum blocking factor (Heppner, 1972; Baldwin, Embleton and Robins, 1973). Although Alexander *et al.* (1969) demonstrated immunoblast cells in the thoracic duct lymph after tumour removal, the effect of tumour excision on *in vitro* tumour growth stimulation by regional lymph node cells has not been described.

In a previous study using the mouse melanoma model, it was reported that spleen cell cytotoxicity was present at the same time as regional lymph node stimulation of tumour growth (Bartholomaeus *et al.*, 1974). The aim of the present experiments was to investigate the changes in host cell mediated and humoral immunity following excision of the tumour at a stage when tumour growth stimulation by the regional lymph nodes was first demonstrable. The same transplantable murine melanoma model was employed except that the tumour cells were inoculated into the footpad and the resulting tumour excised before metastatic spread to the regional lymph nodes occurred.

### MATERIALS AND METHODS

*Mice.*—Inbred female C57BL/6J mice, 6–8 weeks old were used throughout this study.

*Tumour cell lines.*—The B16 melanoma arose spontaneously in a C57BL/6J mouse in 1954 (Green, 1968). It is maintained in this laboratory by serial subcutaneous passage in C57BL/6J mice every third week. This tumour metastasizes to the regional lymph nodes and later to the lungs. However, at the time of tumour excision in this experiment there was no lymph node involvement.

Lewis lung tumour (LLT) is a non-cross-reacting carcinoma which arose spontaneously in a C57BL/6J mouse in 1951 (Sugiura and Stock, 1955). It is maintained in this laboratory in C57BL/6J mice by serial subcutaneous passage.

*Tissue culture cell lines.*—These were established in RPMI 1640 (Grand Island Biological Company, Grand Island, New York, U.S.A.) with 5% foetal calf serum (FCS) and buffered at pH 7·2 according to the formula of Croce (Croce, Koprowski and Eagle, 1972). Cells were discarded after 10 passages *in vitro* and fresh lines established from an *in vivo* passage. Single cell suspensions were obtained by trypsinization with 0·25% trypsin in phosphate buffered saline (PBS).

*Inoculation.*—A single cell suspension of B16 melanoma cells was prepared by passing non-necrotic pieces of tumour tissue through a stainless steel mesh, allowing the larger particles to settle for 5 min, then washing the supernatant cells once in PBS. A concentration of $1 \times 10^7$ viable cells/ml was prepared and 5 $\mu$l was inoculated into the left footpad.

*Lymphoid cells and sera.*—Sera and cells prepared from normal non-tumour bearing control and from tumour bearing groups were pooled from 5 mice on each test day. Animals were exsanguinated under ether anaesthesia by bleeding from the axillary artery and the sera were stored at − 20°C.

Spleen cell suspensions were made by passing the spleens through a stainless steel mesh into Hanks' balanced salt solution (HBSS), allowing the debris to settle for 5 min, and layering the supernatants on to a Ficoll–Hypaque gradient (Penper, Zee and Mickelson, 1968). This was spun at 1000 *g* for 15 min and the mononuclear cells which banded at the interface were removed.

Lymph nodes were dissected from the left popliteal fossa and minced with scissors before aspirating through an 18 gauge needle into HBSS. The debris was allowed to settle for 5 min, after which the supernatant cells were harvested.

Cells from both sources were washed 3 times in HBSS and resuspended at a concentration of $5 \times 10^6$ cells/ml in buffered RPMI 1640.

*Cytotoxicity assay.*—This was based on the method of Takasugi and Klein (1970) using Falcon microtest tissue culture trays (No. 3034 Falcon Plastics, New York, U.S.A.) modified as previously described (Bartholomaeus *et al.*, 1974). Briefly, 5 $\mu$l of lymphoid cell suspensions were added to each well, followed by 5 $\mu$l of B16 melanoma or LLT tissue culture cells at a concentration of $4 \times 10^4$ viable cells/ml, in buffered RPMI 1640 with 10% foetal calf serum. The trays were incubated for 36 h at 37°C with 5% $CO_2$, then inverted for 4 h to allow debris and non-viable cells to fall away from the bottom of each well. The trays were processed by flooding the plates once with PBS, gentle decanting, immersing in methanol for 15 min and subsequently staining with Giemsa stain. The number of target cells remaining in each well was counted under × 80 magnification. B16 melanoma cells were readily identified by their large size and abundant pink staining cytoplasm. LLT cells were smaller and more densely staining but quite distinct from lymphocytes and macrophages. Ten replicates of each culture were prepared.

*Serum cytotoxicity.*—All sera were heat inactivated at 56°C for 30 min, diluted 1 : 2 with culture medium and passed through an 0·45 $\mu$m millipore filter. Normal guinea-pig serum, diluted 1 : 5 in culture medium and sterilized by passage through a millipore filter was used as the source of complement. To determine serum cytotoxicity, 5 $\mu$l of medium containing B16 melanoma cells and 5 $\mu$l of serum were incubated for 1 h at 37°C. 5 $\mu$l of complement was added and the trays were incubated at 37°C under 5% $CO_2$ for 24 h and processed as described above.

*Detection of blocking factors.*—In experiments to test for the presence of serum factors capable of blocking the cytotoxicity of immune spleen cells against B16 melanoma cells, 5 $\mu$l of melanoma cells in culture medium and 5 $\mu$l of serum diluted 1 : 2 in buffered RPMI were incubated at 37°C for 1 h before the addition of 5 $\mu$l of immune spleen cell suspension. The plates were incubated at 37°C under 5% $CO_2$ for 36 h and processed as described previously.

*Experimental design.*—Five animals from both control and tumour bearing groups were processed as described on Days 12, 22, 23, 25, 28 and 34 following inoculation of B16 melanoma. The control group consisted of sex and age-matched non-tumour bearing C57BL/6J mice. As foot amputation of control mice did not significantly change the cytotoxicity of cells from the regional lymph node or spleen in this test system, these animals were used as the source of normal cells and sera.

On Day 22, mice were anaesthetized with Nembutal and the left foot containing the tumour was amputated. At this stage the footpad had doubled in thickness. On Day 23 only sera were collected. The immune spleen cells used in blocking experiments were obtained from mice with a 22-day tumour. Cytotoxicity (relative inhibition of tumour cell growth *in vitro*) was expressed as a percentage, using the formula: $N - T/N \times 100$ (%) where $N$ is the mean number of B16 cells remaining per well with lymphoid cells or serum from control animals and $T$ is the mean number of B16 cells remaining per well with lymphoid cells or serum from animals inoculated with tumour.

### RESULTS

Spleen cell cytotoxicity rose from insignificant levels on Day 12 to 23% on Day 22 (Table I). Three days following tumour excision the cytotoxicity increased from 23 to 28% and was still present at the end of the experiment, 12 days after tumour excision (Day 34). Spleen cells from animals with a 22-day tumour had no significant effect on LLT cells.

The regional lymph node cells at no time showed significant inhibition of tumour cell growth *in vitro* and on Day 22 lymph node cells from tumour bearing mice facilitated the growth of tumour cells (Table I). It was observed that the draining lymph nodes were enlarged at this stage, but there were no B16 cells seen in tissue culture wells if the lymph node cell suspension alone was plated. This eliminated the possibility that metastatic cells in the regional lymph nodes were producing an artefactual tumour growth stimulatory effect. Following tumour excision this effect was lost within 3 days. Regional lymph node cells from mice with a 22-day tumour did not stimulate LLT cells *in vitro*.

TABLE 1.—*Cell Mediated Cytotoxicity*

| Days post-inoculation | Effector cell | Target cell | No. of target cells/well (mean ± S.D.) Normal | Tumour bearing | % Cytotoxicity |
|---|---|---|---|---|---|
| 12 | Spleen cells | B16 | 24·0±4·0 | 22·3±2·2 | 7·0 |
| 22 | Spleen cells | B16 | 16·6±2·8 | 12·7±1·9 | 23·0* |
| 22 | Spleen cells | LLT | 28·6±3·2 | 30·3±4·0 | −6·0 |
| 25 | Spleen cells | B16 | 29·1±5·0 | 20·8±5·1 | 28·5* |
| 28 | Spleen cells | B16 | 30·4±2·9 | 24·8±4·1 | 18·0* |
| 34 | Spleen cells | B16 | 76·9±3·8 | 62·9±6·6 | 19·0* |
| 12 | Lymph node | B16 | 27·6±4·0 | 30·7±3·6 | −11·0 |
| 22 | Lymph node | B16 | 11·1±2·6 | 15·3±3·3 | −33·0* |
| 22 | Lymph node | LLT | 26·4±2·8 | 27·2±3·1 | −3·0 |
| 25 | Lymph node | B16 | 20·7±3·9 | 22·3±4·2 | −8·0 |
| 28 | Lymph node | B16 | 22·8±4·3 | 19·9±4·1 | 13·0 |
| 34 | Lymph node | B16 | 62·0±5·8 | 62·8±5·3 | −1·0 |

* $P < 0·01$ by Student's "$t$" test.

TABLE II.—*Serum Cytotoxicity*

| No. of days post-inoculation when sera collected | No. of B16 cells/well (mean ± S.D.) | % Cytotoxicity |
|---|---|---|
| 0 | 46·7±4·5 | 0 |
| 12 | 36·1±6·6 | 23·0* |
| 22 | 34·1±4·5 | 27·0* |
| 23 | 31·0±5·5 | 33·6* |
| 25 | 29·3±4·6 | 37·2* |
| 28 | 32·6±6·6 | 30·2* |
| 34 | 32·3±4·4 | 30·8* |

* $P < 0·001$ by Students " $t$ " test.

Sera from tumour bearing mice were cytotoxic to melanoma cells compared with sera from control mice (Table II). Serum cytotoxicity increased from 27% pre-operatively to a maximum of 37% 3 days after excision of the tumour. The difference in serum cytotoxicity pre-operatively to that 3 days post-operatively was significant ($P < 0·01$). Serum cytotoxicity was still present 12 days after tumour excision.

Sera from tumour bearing mice blocked the cytotoxicity of immune spleen cells *in vitro* (Table III); 24 h following tumour excision, this blocking effect was not observed and on Day 25, 3 days after tumour excision, the sera increased spleen cell cytotoxicity. This effect was still present 12 days post excision. Normal sera and sera from mice with a 1 cm diameter LLT did not block spleen cell cytotoxicity.

Ten mice from the experimental group were followed for 3 months and no evidence of recurrent tumour was observed.

### DISCUSSION

In this experimental model rapid changes in the host immune response occurred when a growing tumour was excised. Most remarkable was the loss of blocking factor from the serum within 24 h. This was more rapid than previously reported. Baldwin *et al.* (1973) observed blocking to disappear at 3 days; Heppner (1972) showed a slower clearance in which blocking was more consistently absent 10–15 days after tumour excision. A likely explanation for the rapid loss of blocking in this study is that the tumours were excised when they were smaller than those used in the previous studies. Earlier work in this laboratory has shown that there was no spread of tumour to the regional lymph nodes or lungs at this stage of growth. As blocking activity is greatest with large tumour loads, it is thus likely to require a longer time for clearance following tumour excision.

No direct evidence as to the nature of the blocking factor has been elucidated in this study. It would be most unlikely

TABLE III.—*Serum Blocking Factors*

| Effector cells | No. of days post-inoculation when sera collected | No. of B16 cells/well (mean ± S.D.) | % Cytotoxicity | % Blocking† |
|---|---|---|---|---|
| NSP | 0 | 39·8±5·3 | — | — |
| TSP | 0 | 28·3±5·2 | 28·0* | — |
| TSP | 12 | 37·6±4·0 | 5·5* | 81 |
| TSP | 22 | 38·5±2·0 | 3·0* | 90 |
| TSP | 23 | 30·0±3·1 | 25·0 | 10 |
| TSP | 25 | 26·3±4·8 | 34·0 | −17 |
| TSP | 28 | 26·1±5·1 | 34·0 | −17 |
| TSP | 34 | 26·2±3·9 | 34·0 | −17 |
| TSP | LLT sera | 29·2±2·8 | 26·6 | 5 |

NSP = Spleen cells from controls.
TSP = Spleen cells from tumour bearing mice.
* $P < 0·001$ by Student's " $t$ " test.
† Calculation for % blocking:

$$\frac{\%\ \text{cytotoxicity with normal sera} - \%\ \text{cytotoxicity with test sera}}{\%\ \text{cytotoxicity with normal sera}}$$

that the blocking factor was antibody alone, because cytotoxic antibody activity was greatest following excision when no blocking was detectable. The rapid loss of blocking and increase in cytotoxic antibody level following tumour excision could be explained equally well if the blocking factor was an antigen–antibody complex (Sjögren *et al.*, 1971) or tumour antigen, as was first demonstrated by Currie and Basham (1972).

The rise in cytotoxic antibody titre following tumour excision may have been due to a continuing antibody production in the absence of tumour cells which could have bound the antibody or which could have released antigen into the circulation with the formation of antigen–antibody complexes. Because low antibody titres were found in the thoracic duct lymph of tumour bearing animals, Thompson, Steele and Alexander (1973) considered that antigen was released from the tumour cell before being bound by antibody. The rise in antibody titre may enable residual tumour cells to be destroyed either directly by fixing complement or by activating a cell mediated response (Basham and Currie, 1974).

On the basis of *in vivo* studies, Prehn (1972) has postulated that a weak cell mediated immunity may directly stimulate tumour growth. *In vitro* evidence for " immunostimulation " has recently been reported (Medina and Heppner, 1972; Fidler, 1973; Bartholomaeus *et al.*, 1974) in lymph node lymphocytes. Flannery *et al.* (1973), using a rat squamous cell carcinoma, demonstrated regional lymph node cytotoxicity that disappeared with tumour growth, although peripheral lymphocyte cytotoxicity was still present. They attributed the loss of cytotoxicity to increased levels of blocking factor acting in association with an increase in tumour growth. It is possible that a similar mechanism is operative in this experimental model as lymph node stimulation of tumour growth by regional lymph node cells disappeared following the loss of serum blocking factor after the tumour was excised.

It is not considered that the regional lymph node lymphocytes directly stimulate tumour cell growth *in vitro*. A more reasonable explanation is that the regional lymph node cells have attached blocking factor which is not removed by the conventional 3 washings in PBS as previously demonstrated by Currie and Basham (1972) in patients with advanced tumour growth. In tissue culture this blocking factor inhibits lymphocyte reactivity. However, as the control lymph node cells reduce the number of melanoma cells in each tissue culture well over the 36 h incubation period, the net result is an apparent stimulation of tumour growth.

These findings provide *in vitro* evidence to support the work of Alexander *et al.* (1969). They found that the regional lymph node appeared quite active histologically, although there were no increased numbers of immunoblasts in the draining lymph. After tumour excision there was a rapid rise of immunoblasts in the lymph while the regional lymph nodes returned to a normal histological appearance. The suggestion that tumour antigen produced a selective depression on the regional lymph nodes' responsiveness would agree with current views on the nature of blocking (Currie, 1973) and results obtained from this study.

It would appear that the changes in host immunity observed are directly related to removal of the source of tumour antigen, leading to loss of serum blocking and a rise in serum cytotoxicity. Associated with the loss of high concentrations of blocking factor locally, regional lymph node stimulation of tumour growth disappeared. If these *in vitro* findings are directly applicable to the *in vivo* situation, it would suggest that tumour excision confers a number of beneficial effects on the host; the major effect being loss of serum blocking factor in the presence of a continuing cell mediated immunity inhibitory to tumour cell growth.

One of the authors (A. E. B.) is a Medical Postgraduate Scholar of the National Health and Medical Research Council of Australia.

### REFERENCES

Alexander, P., Bensted, J., Delorme, E. J., Hall, J. G. & Hodgett, J. (1969) The Cellular Immune Response to Primary Sarcomata in Rats II. Abnormal Response of Nodes Draining the Tumour. *Proc. R. Soc. Lond.* B, **174**, 237.

Baldwin, R. W., Embleton, M. J. & Robins, R. A. (1973) Cellular and Humoral Immunity to Rat Hepatoma—Specific Antigens Correlated with Tumour Status. *Int. J. Cancer*, **11**, 1.

Bartholomaeus, W. N., Bray, A. E., Papadimitriou, J. M. & Keast, D. (1974) The Immune Response to a Transplantable Malignant Melanoma in Mice. *J. natn. Cancer Inst.* In the press.

Basham, C. & Currie, G. A. (1974) Development of Specific Cell-dependent Antibody during Growth of a Syngeneic Rat Sarcoma. *Br. J. Cancer*, **29**, 189.

Croce, C. M., Koprowski, H. & Eagle, H. (1972) Effect of Environmental pH on the Efficiency of Cellular Hybridization. *Proc. natn. Acad. Sci. U.S.A.*, **69**, 1953.

Currie, G. (1973) The Role of Circulating Antigen as an Inhibitor of Tumour Immunity in Man. In Immunology of Malignancy, Eds. M. Moore, N. W. Nisbet and Mary V. Haigh, *Br. J. Cancer*, **28**, Supp. I, 153.

Currie, G. A. & Basham, C. (1972) Serum Mediated Inhibition of the Immunological Reactions of the Patient to His Own Tumour: a Possible Role for Circulating Antigen. *Br. J. Cancer*, **26**, 439.

Currie, G. A. & Gage, J. O. (1973) Influence of Tumour Growth on the Evolution of Cytotoxic Lymphoid Cells in Rats bearing a Spontaneously Metastasizing Syngeneic Fibrosarcoma. *Br. J. Cancer*, **28**, 136.

Fidler, I. J. (1973) Immunostimulation-Inhibition of Tumour Cell Growth *in vitro* Utilizing Tumour Target Cells Labelled with $^{125}$I-iodo-deoxyuridine. *Immun. Commun.*, **2**, 483.

Flannery, G. R., Chalmers, P. J., Rolland, J. M. & Nairn, R. C. (1973) Immune Response to a Syngeneic Rat Tumour: Development of Regional Node Lymphocyte Anergy. *Br. J. Cancer*, **28**, 118.

Green, E. L. (1968) *Handbook on Genetically Standardized JAX Mice.* Bar Harbor: Times Publishing Co., p. 53.

Hellström, K. E. & Hellström, I. (1971) Some Aspects of the Immune Defense against Cancer: *In vitro* Studies on Animal Tumors. *Cancer, N.Y.*, **28**, 1266.

Hellström, I., Warner, G. A., Hellström, K. E. & Sjögren, H. O. (1973) Sequential Studies on Cell-mediated Tumour Immunity and Blocking Serum Activity in Ten Patients with Malignant Melanoma. *Int. J. Cancer*, **11**, 280.

Heppner, G. H. (1972) *In vitro* Studies on Cell-mediated Immunity following Surgery in Mice Sensitized to Syngeneic Mammary Tumours. *Int. J. Cancer*, **9**, 119.

Klein, G., Sjögren, H. O., Klein, E. & Hellström, K. E. (1960) Demonstration of Resistance against Methylcholanthrene-induced Sarcomas in the Primary Autochthonous Host. *Cancer Res.*, **20**, 1561.

Medina, D. & Heppner, G. (1973) Cell-mediated "Immunostimulation" Induced by Mammary Tumour Virus-free BALB/c Mammary Tumours. *Nature, Lond.*, **242**, 329.

Penper, R. J., Zee, T. W. & Mickelson, M. M. (1968) Purification of Lymphocytes and Platelets by Gradient Centrifugation. *J. Lab. clin. Med.*, **72**, 842.

Prehn, R. T. (1972) The Immune Reaction as a Stimulator of Tumor Growth. *Science, N.Y.*, **176**, 170.

Prehn, R. T. & Main, J. M. (1957) Immunity to Methylcholanthrene-induced Sarcomas. *J. natn. Cancer Inst.*, **18**, 769.

Sjögren, H. O., Hellstrom, I., Bansol, S. C. & Hellstrom, K. E. (1971) Suggestive Evidence that the "Blocking Antibodies" of Tumour Bearing Individuals may be Antigen–Antibody Complexes. *Proc. Nat. Acad. Sci.* U.S.A., **68**, 1372.

Sugiura, K. & Stock, C. C. (1955) Studies in a Tumor Spectrum III. The Effect of Phosphoramides on the Growth of a Variety of Mouse and Rat Tumors. *Cancer Res.*, **15**, 38.

Takasugi, M. & Klein, E. (1970) A Microassay for Cell Mediated Immunity. *Transplantation*, **9**, 219.

Thompson, D. M. P., Steele, K. & Alexander, P. (1973) The Presence of Tumour-specific Membrane Antigen in the Serum of Rats with Chemically Induced Sarcomata. *Br. J. Cancer*, **27**, 27.